# Polyarteritis nodosa involving the hard palate: a case report

**DOI:** 10.1186/1752-1947-7-79

**Published:** 2013-03-18

**Authors:** Eleonora Ortu, Davide Pietropaoli, Mario Baldi, Giuseppe Marzo, Mario Giannoni, Annalisa Monaco

**Affiliations:** 1 Unit of Dentistry; Building Delta 6, Department of Life, Health and Environmental Sciences, University of L’Aquila; San Salvatore Hospital, Via Vetoio, L’Aquila, 67100, Italy

## Abstract

**Introduction:**

Polyarteritis nodosa is a rare disease resulting from blood vessel inflammation (vasculitis), causing damage to organ systems and featuring an extended range of possible symptoms. The cause of polyarteritis nodosa is unknown.

**Case presentation:**

In the present report we describe the presentation and treatment of polyarteritis nodosa involving the hard palate in an 88-year-old Caucasian woman. Clinical and laboratory analyses showed stenosis of the greater palatine artery, which led to necrosis of the affected area. At one year after pharmacological treatment, the lesion has regressed completely.

**Conclusions:**

We successfully treated a case of polyarteritis nodosa via a pharmacological approach, which we describe here.

## Introduction

Polyarteritis nodosa (PAN), also known as Kussmaul disease or Kussmaul-Maier disease, is a vasculitis of the medium and/or small arteries that become swollen and damaged as a result of the attack by rogue immune cells, and the condition may be associated with various atypical presentations [1-3]. The annual incidence of PAN varies between five and nine cases per million [4]. Men are generally more affected than women in a 2:1 ratio, most frequently between the ages of 40 and 60 years. PAN is more common in people with hepatitis B infection [5]. PAN may affect multiple organs, including skin, kidneys, and gastrointestinal tract, as well as the peripheral and central nervous systems [6]. The inflammatory process causes necrosis of cells and structural components of the artery with aneurysms or stenosis formations [7]. PAN often can culminate in necrosis or hemorrhage of the affected organ. Arterial bifurcations are predilected by PAN [7]. This causes a characteristic pattern of aneurysms that Kussmaul and Maier, in their article from 1866, described as being like the apples on the branches of a tree or a wreath of roses [8]. There are no specific laboratory tests for the diagnosis of PAN. The American College of Rheumatology has established criteria for distinguishing PAN from other forms of vasculitis. In the diagnosis of PAN, at least three of the following ten criteria must be considered when radiographic or pathological diagnosis of vasculitis is made [9]: weight loss of 4kg or more; livedo reticularis; testicular pain/tenderness; myalgia or leg weakness/tenderness; mononeuropathy or polyneuropathy; diastolic blood pressure greater than 90mmHg; elevated blood urea nitrogen (BUN) or creatinine level unrelated to dehydration or obstruction; presence of hepatitis B surface antigen or antibody in serum; arteriogram demonstrating aneurysms or occlusions of the visceral arteries; biopsy of small-sized or medium-sized artery containing polymorphonuclear neutrophils.

Here, we report a rare case of PAN that featured necrosis of the hard palate as the main manifestation of the disease. The clinical evidence highlighted a greater palatine artery stenosis, which led to necrosis of the affected area.

## Case presentation

An 88-year-old Caucasian woman, suffering from type 2 diabetes, hypertension (ambulatory blood pressure of 145/90mmHg) and Parkinson's disease was referred to our clinic with a one-week history of a white area on her palate.

**Figure 1:**
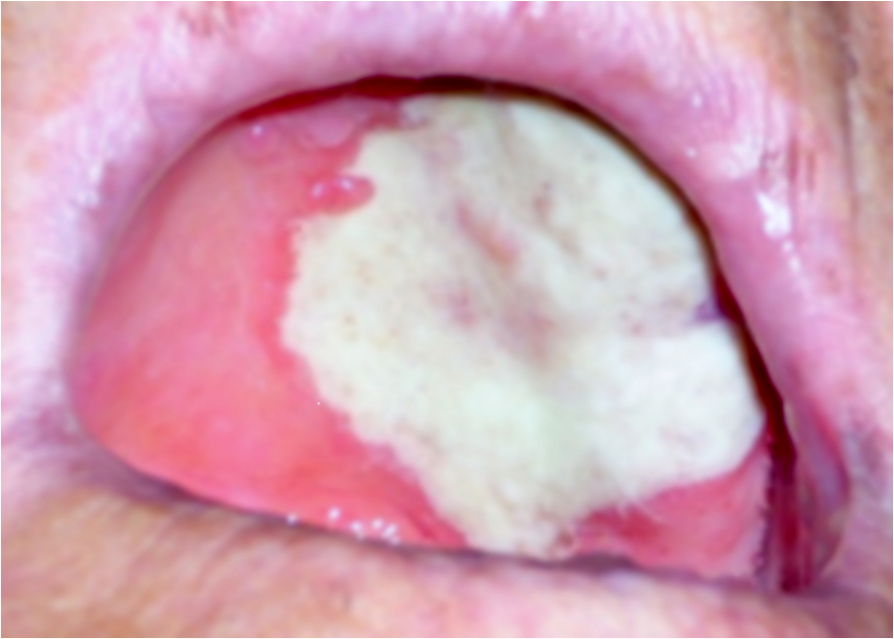
**Palatal lesion at presentation.** On clinical examination, the affected area showed a well demarcated, fibrinous border, corresponding to the vascular zone affected by necrosis.

An oral examination revealed a white area, not raised, occupying more than half of the hard palate. Our patient did not report any pain but her temperature had risen to 38°C during the initial four-day period. A moderate weight loss (5kg), not caused by dietary restriction, was also reported. The results of laboratory tests were as follows: hemoglobin 9.5g/dL, white blood cell count 6,300 cells/L (neutrophils 55 percent, lymphocytes 37 percent, monocytes 5 percent, eosinophils 2 percent, basophils 1 percent), serum creatinine 1.6mg/dL, and erythrocyte sedimentation rate (ESR) 107mm/hour. The results of serum antigen tests for hepatitis B virus (HBV), hepatitis C virus (HCV) and parvovirus B19 were negative. A test for anti-neutrophil cytoplasmic antibodies (ANCAs) was also negative. On clinical examination, the affected area showed a well demarcated fibrinous border, matching the vascular zone affected by necrosis (Figure 1). An incisional biopsy of the peripheral margin of the zone was taken under local anesthesia.

**Figure 2:**
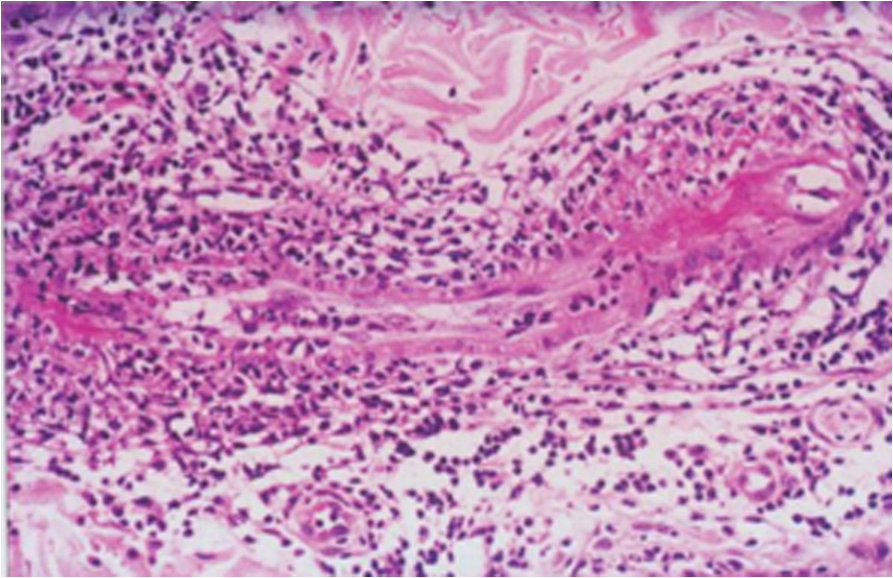
Histological section.

Microscopic study of histological sections revealed that while fibrinoid necrosis involved both lateral extremities of the vascular segment, and that the middle portion was relatively unaffected. The presence of inflammatory infiltrate with variable number of neutrophils, macrophages and scattered lymphocytes was also observed (Figure 2). All these histopathological findings were consistent with a diagnosis of PAN. Also, as suggested by the American College of Rheumatology guidelines, the diagnosis of PAN was made on the basis of six signs that our patient exhibited [9]. It was not possible to perform other studies due to the inability of our patient to move.

According to recent literature [10] laboratory analysis was used to evaluate compete blood counts with potassium, vitamin D3 and calcium. Treatment was started urgently and our patient was treated with oral prednisone at a dosage of 1mg/kg/day, prescribed for three weeks, before being tapered by 5mg every 10 days to a dosage of 0.5mg/kg/day then by 2.5mg every 10 days until a dosage of 15mg/day was obtained and, finally, by 1mg every 10 days to the minimal effective dose or, when possible, until definitive withdrawal. She was also treated with aspirin and warfarin, added to her anti-hypertensive medications, and rinsing with saline solution three to four times at day to clean the affected area. Adjuvant treatment with vitamin D3, calcium and potassium to prevent corticosteroid-induced osteoporosis and cytidine 5'-diphosphocholine (500mg/day) as supportive treatment for Parkinson's disease was also given.

**Figure 3:**
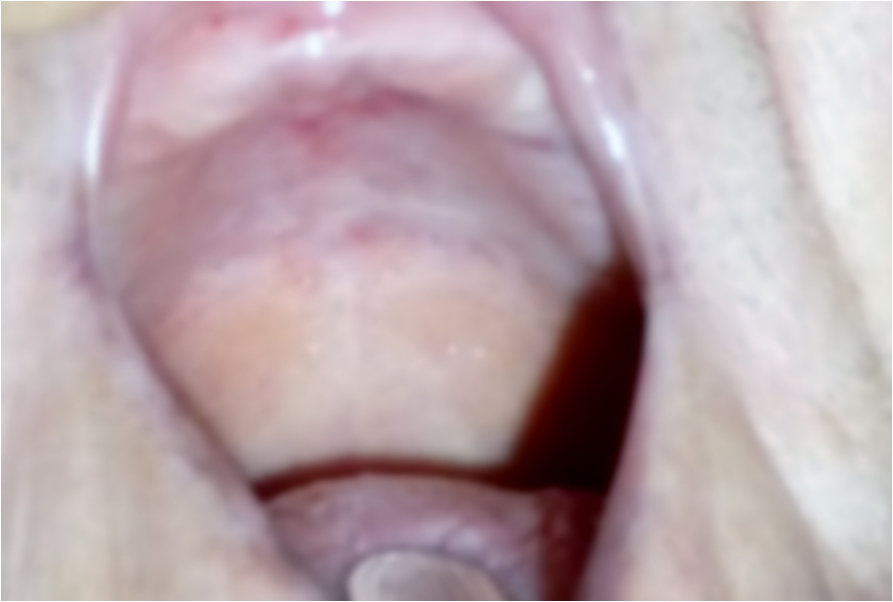
**The lesion two years from onset.** At this time the area of interest is very well vascularized with no recurrences.

After this treatment, the lesion showed improvement and had regressed completely at one year from treatment initiation (Figure 3).

## Discussion

Patients with PAN may present with various symptoms related to vascular involvement within a specific organ. Our patient’s case illustrates a rare and interesting manifestation of PAN with involvement of the hard palate. The authors do not know if the appearance of vasculitis was limited to the palate or whether in the past the pathology had involved other organs.

The pathogenesis of this unusual location is unknown, and to the best of our knowledge has not been described in literature to date.

PAN is a vasculitis that affects small and medium vessels and is characterized by periods of exacerbation and remission periods [7]. This case of PAN showed the involvement of the greater palatine artery, which caused the necrosis of the area of interest. A thorough differential diagnosis is needed to confirm a diagnosis PAN because other forms of vasculitis could lead to an incorrect diagnosis. No specific tests are known for diagnosis of PAN, but the American College of Rheumatology suggests 10 clinical criteria including blood profiles of antibodies and angiography and histology [11]. Patients may present with a variety of symptoms, the most common of which are fever (25 percent to 56 percent), neuropathy (77 percent to 92 percent) and weight loss (63 percent to 85 percent) [12]. The differential diagnosis should include other systemic autoimmune diseases (systemic lupus erythematosus, severe rheumatoid arthritis and others) or marked fever and weight loss in tumor cases [1]. Corticosteroids are usually the first-line treatment drugs and a response is usually noted within three months. Cyclophosphamide has shown a better outcome in patients with severe disease as well as those refractory to steroid treatment. Although there is no clear survival benefit when steroids are used in combination with cyclophosphamide, the relapse rate was reduced significantly [4].

In our patient’s case, PAN was not treated with cyclophosphamide as use of corticosteroids significantly reduced the lesion, restoring proper blood circulation in the affected area. Our patient was also treated with anti-osteoporotic drugs to avoid consequent bone problems. The drugs already prescribed to our patient for the control of diabetes and Parkinson’s disease were not modified with regard to dosages. Only aspirin and warfarin were added to the anti-hypertensive medications.

## Conclusions

The present report describes the successful treatment of PAN via a pharmacological approach. The clinical case presented is a very rare event and to the best of our knowledge has not been described in the literature previously. At two years after the appearance of the lesion, the area of interest is very well vascularized and there have been no recurrences. Routine blood tests and biopsies are essential for diagnosis and to avoid delays in treatment and possible organ damage.

## Consent

Written informed consent was obtained from the patient for publication of this case report and any accompanying images. A copy of the written consent is available for review by the Editor-in-Chief of this journal.

## Competing interests

The authors declare that they have no competing interests.

## Authors’ contributions

AM, MG and MB analyzed and interpreted the data from our patient regarding the blood tests and the disease. AM, GM, MG and MB made the diagnosis of PAN and established the treatment. EO and DP were the major contributors in writing the manuscript. All authors read and approved the final manuscript.
